# Efficacy of sacituzumab govitecan in triple-negative breast cancer with hepatic visceral crisis: a case report

**DOI:** 10.3389/fonc.2026.1790357

**Published:** 2026-02-27

**Authors:** Jiaqi Ye, Jiaang Ye, Huijuan He, Zhongyu Jiang, Yan Wang, Li Wang

**Affiliations:** 1 Department of Radiotherapy, The Quzhou Affiliated Hospital of Wenzhou Medical University, Quzhou People’s Hospital, Quzhou, Zhejiang, China; 2 Department of Stomatology, Jining Medical University, Jining, Shandong, China

**Keywords:** antibody-drug conjugate, hepatic visceral crisis, partial response, sacituzumab govitecan, triple-negative breast cancer

## Abstract

Triple-negative breast cancer (TNBC) is recognized as the most aggressive molecular subtype of breast cancer (BC). The liver is a common site of distant metastasis in BC, and hepatic involvement is associated with accelerated therapeutic resistance and increased mortality risk. Although sacituzumab govitecan (SG) has shown favorable clinical efficacy in the treatment of BC with liver metastasis in the ASCENT randomized controlled trial and real-world studies, its application value in hepatic visceral crisis (HVC) remains inadequately investigated. Herein, we report the clinical course and outcomes of a TNBC patient with diffuse liver metastasis. Following two cycles of SG treatment, the patient achieved significant remission of liver lesions, with a progression-free survival (PFS) of 16.3 months. This case report highlights the necessity of conducting further in-depth studies to explore the application of SG in BC patients with visceral crisis (VC).

## 1 Introduction

TNBC defined by the absence of estrogen receptor (ER), progesterone receptor (PR), and human epidermal growth factor receptor 2 (HER2) expression, is recognized as the most aggressive molecular subtype of breast cancer (BC), accounting for 10–15% of all BC cases. Compared with hormone receptor (HR)-positive or HER2-positive tumors, TNBC is associated with significantly shorter disease-free survival (DFS) and overall survival (OS) (1). Population-based studies indicate that more than 50% of patients with localized TNBC who receive standard multimodal therapy experience disease recurrence within 5 years of initial diagnosis (2, 3). The liver is a common site of distant metastasis in BC, and hepatic involvement is associated with accelerated therapeutic resistance and increased mortality risk. Historical studies report a median survival of 3–15 months after the detection of liver-only or liver-dominant metastases, with a 5-year survival rate as low as 8.5% (3, 4).

Trophoblast cell-surface antigen 2 (Trop-2), a transmembrane epithelial glycoprotein overexpressed in 80–90% of TNBC, is associated with accelerated tumor growth, shortened survival and poor prognosis, thus serving as a validated therapeutic target (5–7). SG is an antibody–drug conjugate(ADC) consisting of a humanized anti-Trop-2 monoclonal antibody, a cleavable hydrolysable linker and SN-38, the active metabolite of irinotecan that inhibits topoisomerase I. In the randomized phase III ASCENT trial (NCT02574455), SG was compared with physician’s choice of standard chemotherapy (TPC) in patients with metastatic TNBC who had progressed after ≥ two prior systemic regimens SG significantly prolonged both PFS and OS. The median PFS was 5.6 months versus 1.7 months with TPC (hazard ratio [HR] 0.41; 95% confidence interval [CI] 0.32–0.52; p < 0.001), and the median OS was 12.1 months versus 6.7 months (HR 0.48; 95% CI 0.38–0.59; p < 0.001). These data have established SG as the standard second-line treatment for metastatic TNBC (8).

When metastatic tumor burden leads to rapid deterioration of organ function, the condition is termed “ VC “, which occurs in approximately 10%–15% of patients diagnosed with advanced breast cancer (9). HVC in breast cancer specifically refers to a critical clinical state characterized by rapid multi-organ dysfunction induced by tumor burden; its definition remains imprecise and largely depends on individual clinical judgment.Liver-specific VC is defined as a rapid elevation of bilirubin to >1.5 times the upper limit of normal, with the exclusion of Gilbert’s syndrome or biliary tract obstruction (9). This condition is associated with an extremely poor prognosis, with a mortality rate of 90% even after standard treatment (10). Notably, VC is a common exclusion criterion in clinical trials, leaving the optimal treatment strategy in a gray area. Furthermore, the administration of anticancer therapy presents particular challenges in patients with extensive liver metastases. Currently, data on systemic treatment for TNBC complicated by VC remain limited.

In this report, we present the clinical course and outcomes of a patient with metastatic, early-recurrent TNBC and HVC who achieved a remarkable response to SG as third-line treatment.

## 2 Case description

### 2.1 Initial diagnosis and curative-intent treatment

A 43-year-old female patient with no relevant medical history and no family history of malignant tumors was diagnosed with TNBC accompanied by lymph node metastasis in August 2020. Following 8 cycles of neoadjuvant chemotherapy, she underwent modified radical mastectomy of the left breast in January 2021.

Postoperative pathological examination showed: invasive ductal carcinoma of the breast, World Health Organization (WHO) grade III, Miller-Payne (MP) grade II, tumor size 4 cm × 2.5 cm × 2 cm, with detectable vascular and lymphatic space tumor emboli; 1 left axillary lymph node was positive for metastatic carcinoma. Immunohistochemical (IHC) results were as follows: ER (-), PR)(-), HER-2)(-), and Ki-67 proliferation index (70%).

Subsequent to surgery, the patient received adjuvant radiotherapy with a total dose of 50 Gy in 25 fractions. After the completion of radiotherapy, she was started on oral capecitabine for adjuvant chemotherapy, and the treatment was well-tolerated without significant adverse events.

### 2.2 Development of metastatic disease

In September 2021, chest computed tomography (CT) revealed lung metastases. The patient was then treated with albumin-bound paclitaxel at a dose of 260 mg/m² every 3 weeks, completing a total of 6 cycles. Follow-up evaluation showed stable pulmonary lesions. In May 2022, cranial magnetic resonance imaging (MRI) indicated brain metastases, for which she received brain lesion-targeted radiotherapy combined with pembrolizumab immunotherapy.

In April 2023, a chest CT scan demonstrated enlargement of the pulmonary metastases. Due to the patient’s refusal of chemotherapy, she was initiated on pulmonary metastasis-targeted radiotherapy combined with pembrolizumab immunotherapy. In September 2023, progression of intracranial lesions was observed, and cisplatin plus gemcitabine combined with pembrolizumab was administered. After 2 cycles of treatment, follow-up assessment showed a reduction in both lung and brain metastases compared with the baseline, which was evaluated as a partial response (PR).

However, the patient developed significant chemotherapy-related adverse events and refused further chemotherapy. She was then switched to maintenance therapy with oral capecitabine combined with pembrolizumab. Reassessment in January 2024 showed regression of brain metastases and stable lung disease. The patient declined continued oral capecitabine and was therefore switched to pembrolizumab monotherapy.

### 2.3 Onset of hepatic visceral crisis

In April 2024, the patient presented with abdominal distension, and had an ECOG performance status (ECOG PS) of 1. Liver MRI revealed diffuse multiple metastatic lesions in the liver. Ultrasound-guided liver biopsy confirmed poorly differentiated carcinoma with necrosis, which was deemed to be of breast origin. IHC results were as follows: GS (focal+), CK18 (-), CD34 (vascular+), Hepatocyte (-), GPC-3 (-), Ki-67 (60%+), GATA3 (+), P120 (membrane+), E-Cadherin (+), Her-2 (-), PR (-), ER (-); whole-exome sequencing of BRCA1/2 was negative. The corresponding laboratory data are summarized in (**Table 1**). The serum bilirubin level of this patient exceeded 1.5-fold the upper limit of normal, consistent with the definition of HVC established by ESO-ESMO.

**Table 1:** Laboratory results of the patient at time of presentation with HVC.

Test	Units	Reference	Numerical value
Total bilirubin	umol/L	5.1-20.5	40.6
Direct bilirubin	umol/L	0.0-6.8	25.5
AST	U/L	4.0-48.0	178
ALT	U/L	4.0-42.0	31.1
ALP	U/L	34.0-121.0	669.6
GGT	U/L	4.0-60.0	980.9
Albumin	g/L	35.0-53.0	30.2
Creatinine	umol/L	35.0-115.0	59.4
INR		0.91-1.22	1.06

AST, Aspartate Aminotransferase; ALT, Alanine Aminotransferase; ALP, Alkaline Phosphatase; GGT, Gamma-Glutamyl Transferase; INR, International Normalized Ratio.

### 2.4 Treatment with SG and clinical outcome

**Figure 1:**
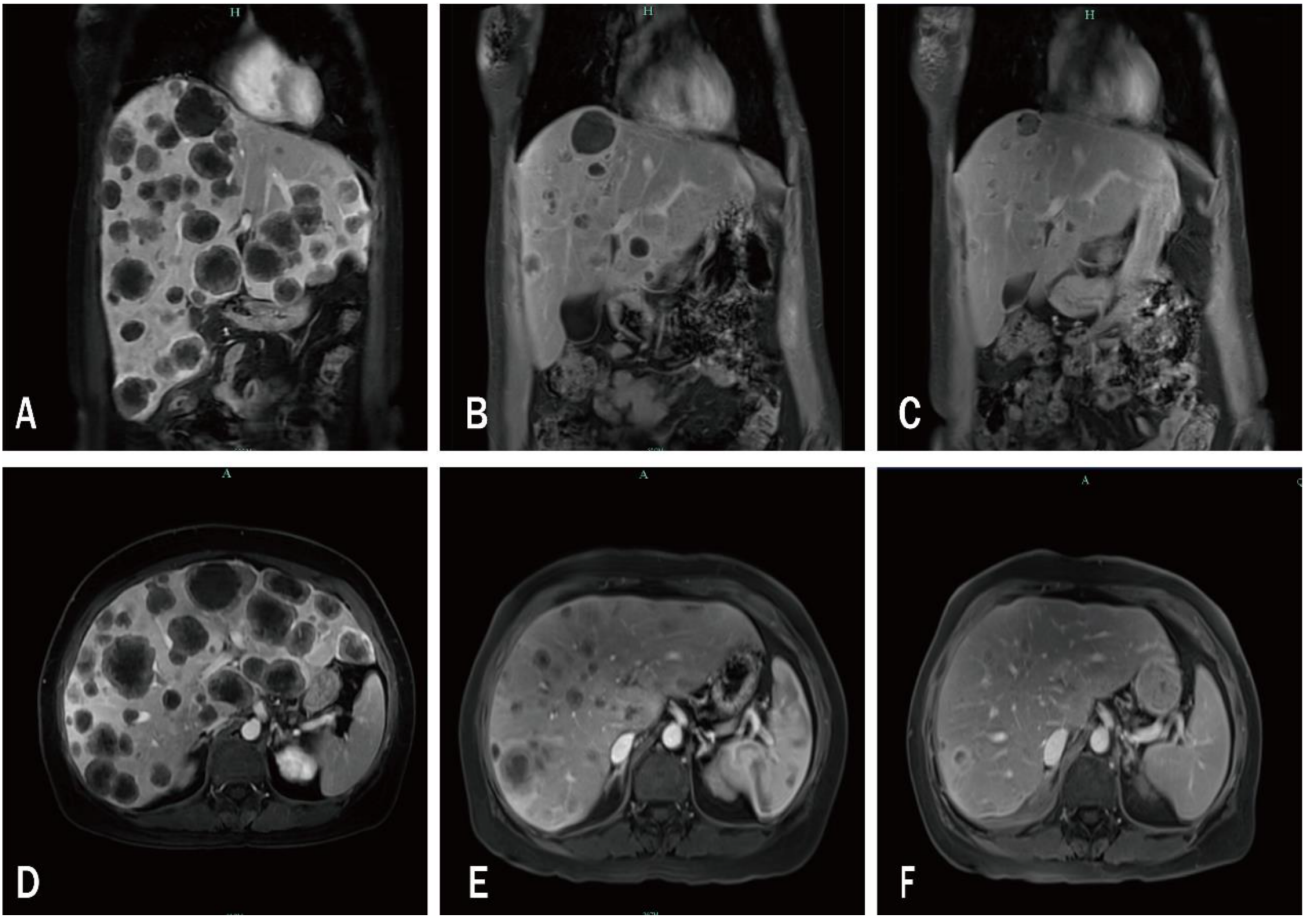
Serial T1-weighted magnetic resonance images of the upper abdomen. **(A, D)** baseline scan (9 April 2024) showing multiple intra-hepatic metastases; **(B, E)** follow-up after two treatment cycles (26 May 2024) demonstrating marked tumor regression; **(C, F)** subsequent follow-up (31 July 2024) revealing continued reduction in liver metastatic burden.

**Figure 2:**
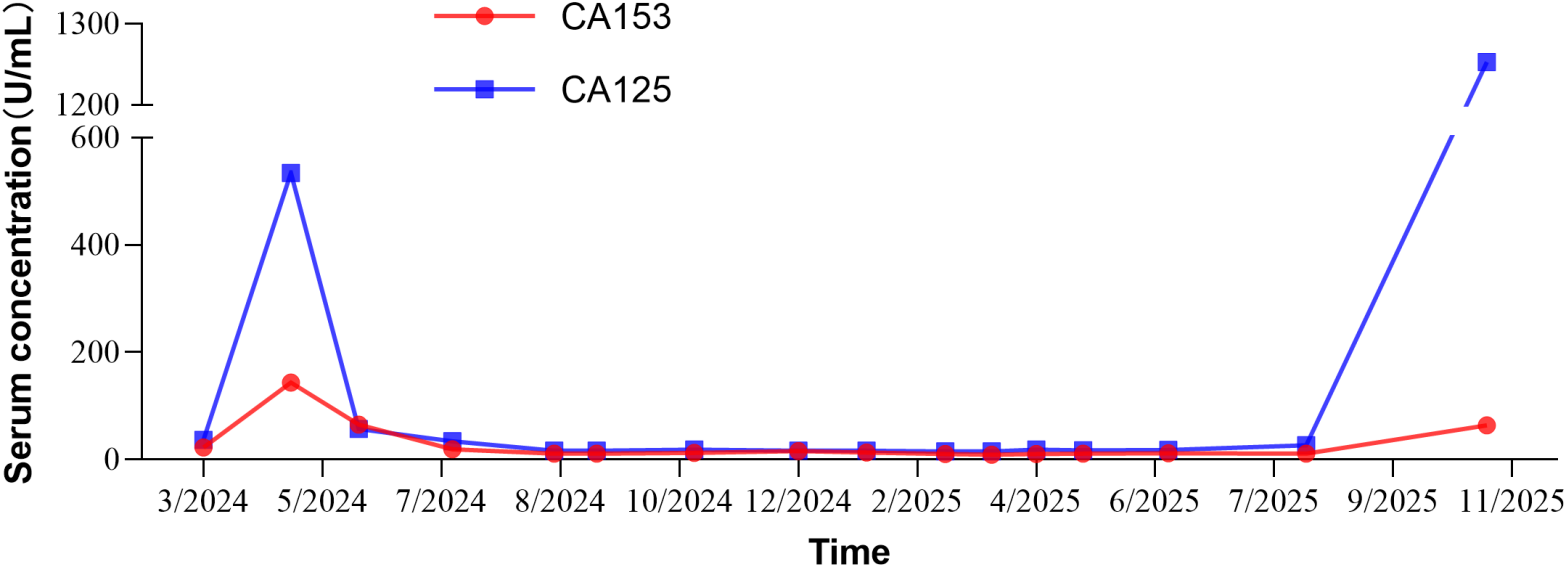
Temporal profiles of serum CA125 and CA153 concentrations.

The patient was then administered two cycles of SG (10mg/kg, 540 mg, d1, 8 q3w) in combination with pembrolizumab. Follow-up imaging performed on 26 May 2024 revealed a marked reduction in intrahepatic metastatic tumor burden (**Figure 1**), concomitant with substantial decreases in serum CA125 and CA153 concentrations (**Figure 2**).

During the treatment course, the patient developed recurrent symptoms of frequent urination, urinary urgency, and dysuria, accompanied by elevated serum creatinine levels, raising suspicion for immune-mediated cystitis. Pembrolizumab therapy was therefore discontinued, and the patient continued treatment with SG monotherapy.

**Figure 3:**
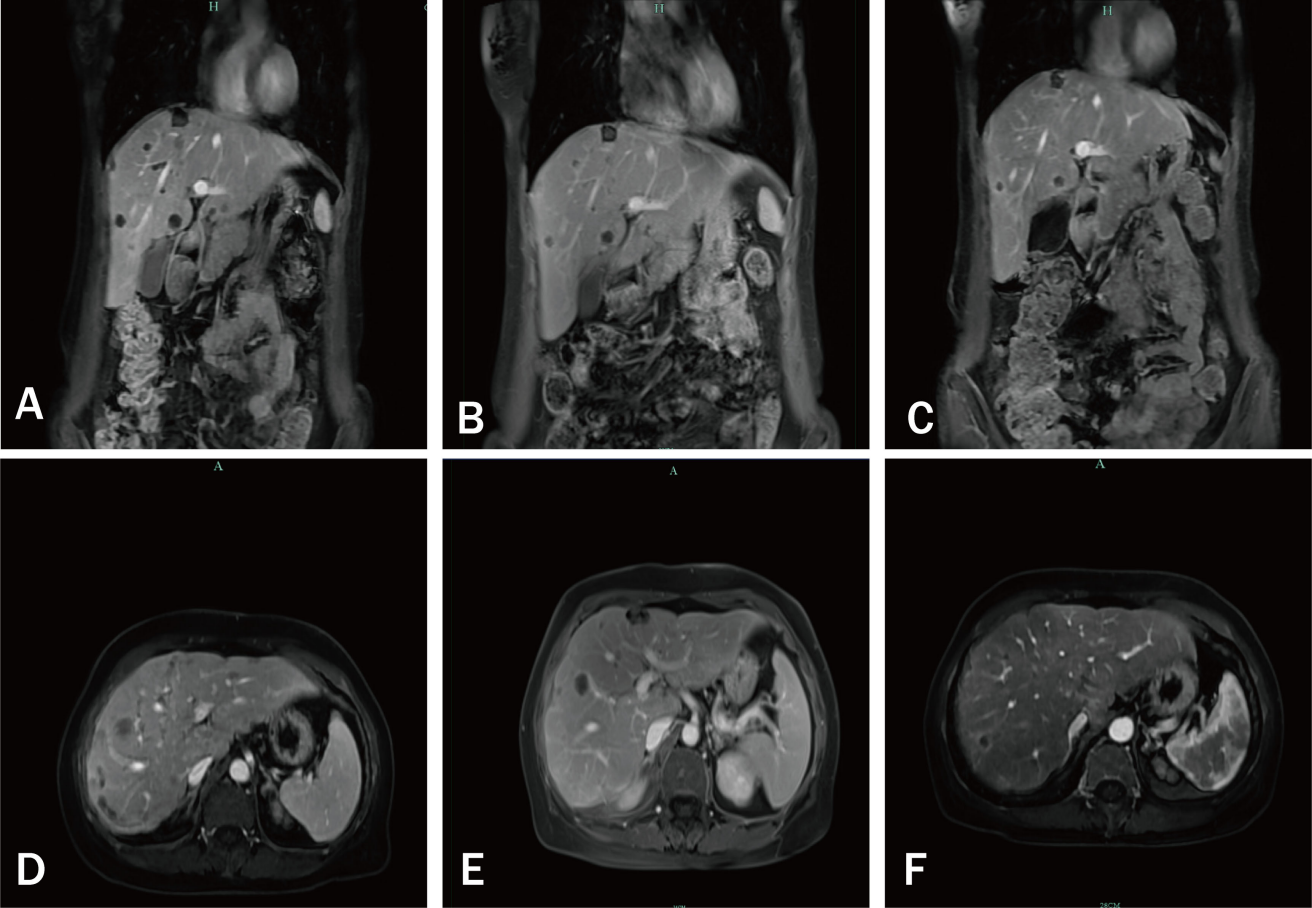
Serial contrast-enhanced T1-weighted magnetic resonance images of the abdomen demonstrating temporal evolution of hepatic lesions. **(A, D)** 16 September 2024; **(B, E)** 22 November 2024; **(C, F)** 6 February 2025.

**Figure 4:**
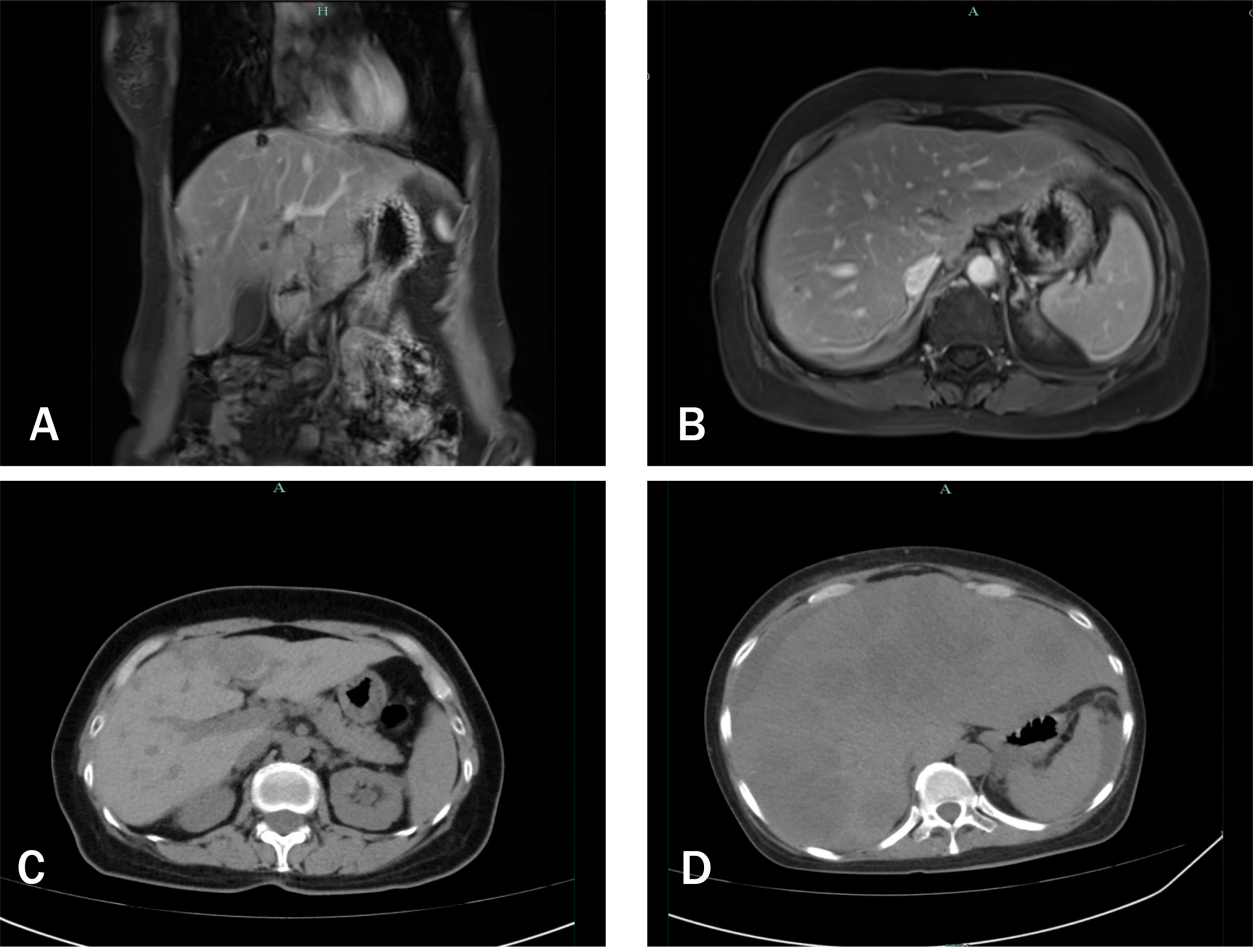
Serial imaging of hepatic metastases. **(A, B)** Contrast-enhanced T1-weighted MRI (13 June 2025) showing sustained reduction in intrahepatic tumor burden. **(C)** Contrast-enhanced CT (16 August 2025) demonstrating early regrowth of liver metastases. **(D)** CT (8 November 2025) revealing diffuse, multifocal hepatic progression consistent with HVC.

Subsequent regular follow-up evaluations showed sustained shrinkage of the lesions (**Figures 3**, **4A, B**). The patient expressed satisfaction with both the treatment efficacy and her quality of life.

However due to financial constraints, the patient opted to extend the dosing interval and reduce the treatment frequency. An abdominal CT scan performed on August 16, 2025, showed enlargement of the hepatic lesions compared with previous imaging findings (**Figure 4C**).

**Figure 5:**
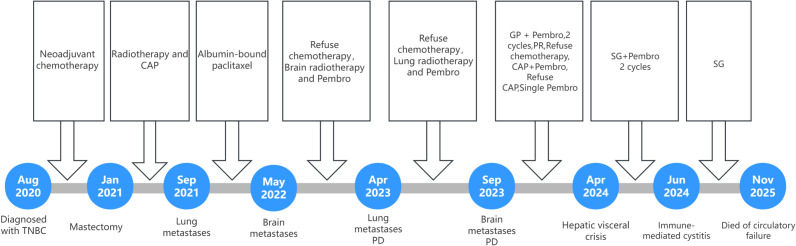
Clinical timeline of the case. CAP, Capecitabine; Pembro, Pembrolizumab; GP, Gemcitabine plus cisplatin; PD, Progressive Disease;.

The patient then initiated self-administered oral traditional Chinese medicine therapy. In November 2025, the patient presented with ascites, severe anemia, fever, and anuria. Follow-up CT scan demonstrated significant progression of the hepatic lesion and massive ascites (**Figure 4D**). Abdominal paracentesis yielded non-clotting blood, suggesting rupture and hemorrhage of the hepatic metastatic tumor. The patient ultimately died of circulatory failure in November 2025. **Figure 5** presents the clinical timeline of the case.

## 3 Discussion

SG is a novel ADC consisting of a humanized anti-Trop-2 antibody (RS7) conjugated to SN38 (11, 12). SN38 selectively binds to topoisomerase I and abrogates the religation of topoisomerase I-mediated single-strand DNA breaks, ultimately culminating in DNA damage, subsequent apoptosis, and eventual cell death. Beyond the direct cytotoxic effects exerted by SN38, SG further elicits antibody-dependent cellular cytotoxicity (ADCC) (13).

The phase III ASCENT trial led to the approval of SG (10 mg/kg, d1, 8 q3w) in patients with advanced or metastatic TNBC who have received ≥2 prior systemic therapies (14). In the subgroup analysis of this trial, patients with liver metastases constituted 43% of the total study population (42% in the SG group vs. 44% in the treatment of physician’s choice [TPC] group). The final results demonstrated that, in patients with liver metastases, SG achieved a statistically significant improvement in both PFS (hazard ratio [HR], 0.42; 95% confidence interval [CI], 0.31–0.57) and OS (HR, 0.53; 95% CI, 0.40 to 0.70) compared with TPC. The magnitude of clinical benefit was analogous to that observed in the overall study population, indicating that the presence of liver metastases did not significantly alter the therapeutic effect of SG (15). However, it is noteworthy that this trial excluded patients with severe visceral involvement or HVC. The efficacy of SG observed in this patient with HVC is consistent with and extends the findings of the phase III ASCENT trial. Specifically, our case addresses a critical gap in the literature by providing real-world evidence of SG’s antitumor activity in TNBC patients with HVC. This patient experienced a significant reduction in intrahepatic metastatic lesions after only two cycles of SG combined with pembrolizumab, with sustained lesion shrinkage noted during subsequent SG monotherapy. Ultimately, HVC was reversed, and the PFS was prolonged to 16.3 months; throughout the entire treatment period, the patient maintained a favorable quality of life.

Regrettably, owing to the high cost of the drugs, the patient opted to extend the medication interval and reduce the administration frequency, which ultimately resulted in disease progression. This observation aligns with preclinical and clinical evidence highlighting that optimal dosing of ADCs is critical for sustaining therapeutic efficacy; deviations from the recommended regimen may compromise drug exposure and thereby diminish antitumor activity.

Notably, financial barriers to cancer treatment represent a prominent global challenge. A cost-effectiveness analysis conducted from the perspective of a third-party US payer revealed that SG was not a cost-effective treatment option relative to single-agent chemotherapy in patients with pretreated, unresectable or metastatic HR+/HER2− breast cancer. Specifically, SG treatment increased the quality-adjusted life years (QALYs) by 0.217 years compared with chemotherapy, yielding an incremental cost-effectiveness ratio of US$612, 772 per QALY—exceeding the widely accepted willingness-to-pay (WTP) threshold of US$150, 000 per QALY (16). Additionally, a study conducted in China indicated that the price of SG was substantially higher than that of chemotherapy, which emerged as a key determinant of cost-effectiveness. Therefore, targeted measures—including substantial price reductions, robust patient assistance programs, and optimized dosing strategies—are warranted to render SG a cost-effective therapeutic option for TNBC treatment (17, 18).

Building on the aforementioned context and the charitable drug donation program for pembrolizumab, the patient in this case initially received two cycles of pembrolizumab in combination with SG, which yielded robust therapeutic efficacy. Unfortunately, prolonged administration of pembrolizumab induced immune-related cystitis, prompting the discontinuation of this agent, the patient continued to exhibit favorable therapeutic responses during subsequent SG monotherapy. However, several challenges remain in the use of SG. As reported in a study by Kumari et al. (19), the efficacy of SG may be different in patients treated with different types of drugs owing to their different mechanisms of action. The genetic profiling of patients specifically for cell cycle or DNA repair genes will also be helpful in evaluating the effect of SG in TNBC patients.

Mechanistically, pembrolizumab competitively binds to programmed cell death protein 1 (PD-1), blocks the PD-1/programmed death-ligand 1 (PD-L1)/programmed death-ligand 2 (PD-L2) signaling pathway, alleviates tumor-mediated T-cell suppression, restores and enhances the antitumor functionalities of CD8^+^ cytotoxic T lymphocytes and CD4^+^ helper T cells, and concurrently facilitates the generation of memory T cells—ultimately culminating in a sustained antitumor immune response (20).

The effectiveness and safety of pembrolizumab in combined with SG remain under active investigation. As a global, multicenter, randomized Phase III trial, the AsK-04/KEYKEYNOTE -D19 study enrolled 443 patients with PD-L1 positive (CPS≥10) locally advanced or metastatic TNBC to compare the efficacy of SG plus pembrolizumab versus standard chemotherapy plus pembrolizumab. Preliminary findings demonstrated that the median progression-free survival (mPFS) in the SG plus pembrolizumab group reached 11.2 months, which was significantly superior to the 7.8 months observed in the chemotherapy plus pembrolizumab group (HR = 0.65, P<0.001), representing a 35% reduction in the risk of disease progression or death. The objective response rate (ORR) was 59% vs 53%, and the median duration of response was prolonged to 16.8 months (8.6 months in the chemotherapy combination group). While OS data remain immature, a trend toward clinical benefit has been observed, and the safety profile was consistent with the known characteristics of each monotherapy. Notably, this study is the first to confirm that SG combined with immune checkpoint inhibitors (ICIs) is superior to chemotherapy combined with ICIs, and is anticipated to become a new standard of care for first-line treatment of PD-L1-positive mTNBC (21). Results from another phase II trial (LBA1004) in patients with metastatic hormone receptor (HR)-positive, HER2-negative breast cancer showed that the combination of SG plus pembrolizumab did not yield a statistically significant difference in PFS compared with gemcitabine plus capecitabine (22). Therefore, the efficacy and safety of pembrolizumab combination with SG still require further in-depth research and exploration.

Patients with HVC represent an urgent and unmet clinical need, as they are frequently excluded from clinical trials, —primarily due to their rapid disease progression and poor prognosis. To the best of our knowledge, this is the first case report documenting the efficacy of SG in HVC. A significant and sustained clinical response was observed after the first cycle of treatment, with a remarkable PFS of 16.3 months, during this period, the patient achieved complete clinical recovery and maintained an active quality of life. This case underscores the critical need for further dedicated research focusing on the management of HVC.

## Data Availability

The original contributions presented in the study are included in the article/supplementary material. Further inquiries can be directed to the corresponding authors.
